# Prevalence and Microbial Association of Onychomycosis in Patients with Diabetic Foot Ulcers: A Retrospective Observational Study

**DOI:** 10.7759/cureus.115068

**Published:** 2026-08-24

**Authors:** Abishek C R, Sunil Kumar, Namitha

**Affiliations:** 1 Department of General Surgery, Cosmopolitan Hospital, Thiruvananthapuram, IND

**Keywords:** diabetic foot ulcer, fungal culture, fungal nail infection, microbial association, onychomycosis, polymicrobial infection, potassium hydroxide microscopy, prevalence

## Abstract

Background: Onychomycosis is a common fungal nail infection among individuals with diabetes mellitus and may contribute to diabetic foot complications by altering the local microbial environment and increasing the risk of secondary bacterial infection. This study aimed to determine the prevalence of onychomycosis and evaluate its association with clinical and microbiological characteristics among patients with diabetic foot ulcers.

Methodology: This retrospective observational study included 226 adult patients with diabetic foot ulcers treated at a tertiary referral center in South Kerala, India. Clinical records were reviewed to obtain demographic, clinical, and microbiological data. Onychomycosis was diagnosed based on compatible clinical findings with microbiological confirmation using potassium hydroxide microscopy and/or fungal culture. Categorical variables were summarized using frequencies and percentages. Univariate binary logistic regression was performed to identify factors associated with onychomycosis.

Results: Of the 226 patients included in the study, 85 (37.6%) had confirmed onychomycosis. Among patients with onychomycosis, 35 (41.2%) were aged 61-70 years, 54 (63.5%) were male, and 60 (70.6%) had diabetes duration >10 years. Poor glycemic control (HbA1c >9%) was observed in 43 (50.6%) patients, while peripheral neuropathy was present in 73 (85.9%). Polymicrobial ulcer cultures were identified in 44 (51.8%) patients with onychomycosis. Gram-negative bacilli were isolated in 49 (57.6%) patients, *Pseudomonas aeruginosa* in 30 (35.3%), and osteomyelitis was present in 35 (41.2%). On univariate binary logistic regression analysis, diabetes duration >10 years (OR: 2.30, 95% CI: 1.24-4.28; p=0.008), peripheral neuropathy (OR: 2.67, 95% CI: 1.33-5.34; p=0.005), polymicrobial infection (OR: 2.26, 95% CI: 1.30-3.91; p=0.004), *Pseudomonas aeruginosa* isolation (OR: 2.10, 95% CI: 1.14-3.86; p=0.017), and osteomyelitis (OR: 2.38, 95% CI: 1.33-4.26; p=0.003) were significantly associated with onychomycosis.

Conclusions: More than one-third of patients with diabetic foot ulcers had concomitant onychomycosis. Longer duration of diabetes, peripheral neuropathy, polymicrobial infection, Pseudomonas aeruginosa isolation, and osteomyelitis were significantly associated with onychomycosis. Routine nail examination and timely identification of fungal nail infection may complement comprehensive diabetic foot assessment and help identify patients at increased risk of complicated foot infections.

## Introduction

Diabetic foot ulcers are a major complication of diabetes mellitus and are associated with substantial morbidity, prolonged hospitalization, increased healthcare costs, and lower-extremity amputation [1]. Their development is multifactorial, resulting from the combined effects of peripheral neuropathy, peripheral arterial disease, impaired immunity, foot deformities, and repetitive trauma. Secondary infection further complicates ulcer healing, increasing the risk of deep tissue involvement, osteomyelitis, and limb loss [2].

Onychomycosis is a chronic fungal infection of the nail caused by dermatophytes, non-dermatophyte molds, or yeasts. Patients with diabetes are particularly susceptible because of impaired peripheral circulation, peripheral neuropathy, repeated nail trauma, and altered immune function [3]. Progressive nail dystrophy, including nail thickening, onycholysis, and subungual hyperkeratosis, may increase local pressure, cause periungual skin injury, and facilitate bacterial entry, thereby contributing to diabetic foot complications [4]. Despite its potential clinical significance, onychomycosis is frequently overlooked during routine diabetic foot assessment, which primarily focuses on vascular insufficiency, peripheral neuropathy, and ulcer management.

The microbiology of diabetic foot ulcers is predominantly bacterial, with Staphylococcus aureus, *Pseudomonas aeruginosa*, and members of the *Enterobacteriaceae* family among the most commonly isolated pathogens [5]. Chronic ulcers frequently harbor polymicrobial communities, and their microbial composition is influenced by ulcer duration, prior antibiotic exposure, and biofilm formation. In contrast, fungal infections, particularly onychomycosis, are not routinely evaluated despite growing evidence suggesting that they may alter the local microbial environment and contribute to the complexity of diabetic foot infections [4-8].

Although several studies have reported an increased prevalence of onychomycosis among patients with diabetes mellitus, limited evidence is available regarding its relationship with the microbiological profile of diabetic foot ulcers, particularly in the Indian population [7]. Therefore, this study aimed to determine the prevalence of onychomycosis among patients with diabetic foot ulcers and evaluate its association with demographic characteristics, clinical risk factors, and ulcer microbiology. A better understanding of these associations may facilitate early identification of high-risk patients and support a more comprehensive approach to diabetic foot management.

## Materials and methods

Study design and setting

This retrospective observational study was conducted at Cosmopolitan Hospital, a tertiary referral healthcare center in Thiruvananthapuram, Kerala, India. Clinical records of patients with diabetic foot ulcers who underwent nail examination and microbiological evaluation during the study period were reviewed. The study utilized existing hospital records to assess the prevalence of onychomycosis and its association with ulcer microbiology and clinical characteristics.

Study population

The study included adult patients (≥18 years) with a confirmed diagnosis of type 1 or type 2 diabetes mellitus who presented with one or more diabetic foot ulcers. A total of 226 eligible patients with available clinical, nail examination, and microbiological records were included in the analysis.

Eligibility criteria

Patients were included if they were aged 18 years or older, had a confirmed diagnosis of diabetes mellitus, presented with at least one diabetic foot ulcer, had documented nail examination findings with confirmation or exclusion of onychomycosis, and had available microbiological culture results from ulcer specimens (pus and/or deep tissue).

Patients were excluded if they had non-diabetic ulcers (e.g., traumatic or vasculitic ulcers), critical limb ischemia that precluded adequate ulcer assessment, incomplete nail examination or microbiological records, or had received recent systemic antifungal therapy that could interfere with the diagnosis of onychomycosis.

Data collection

Data were extracted from electronic and paper medical records using a standardized data collection form. Demographic variables included age and sex. Clinical variables included duration of diabetes mellitus, glycemic control assessed by glycated hemoglobin (HbA1c), presence of peripheral neuropathy, peripheral arterial disease, foot deformity, history of trauma, ulcer location, Wagner grade, osteomyelitis, previous diabetic foot ulcer episodes, and treatment interventions. Nail characteristics recorded included discoloration, nail thickening, onycholysis, subungual debris, nail dystrophy, and hypertrophic nails.

Diagnosis of onychomycosis

Onychomycosis was diagnosed based on compatible clinical nail findings together with microbiological confirmation. Laboratory confirmation consisted of a positive potassium hydroxide mount demonstrating fungal elements and/or a positive fungal culture obtained from nail or tissue specimens. As this was a retrospective, record-based study, the diagnosis was based on clinical and microbiological findings documented in hospital records. Fungal microscopy and culture were not performed in all patients; therefore, patients were classified according to the available documented diagnostic evaluation. Patients were subsequently classified into onychomycosis-positive and onychomycosis-negative groups.

Microbiological evaluation

Deep tissue or pus specimens obtained during wound debridement were processed according to the standard microbiological laboratory protocols followed at the study center for culture and antimicrobial susceptibility testing. The microbiological evaluation included culture and identification of bacterial isolates in accordance with routine laboratory procedures. Culture results were categorized as no growth, monomicrobial growth, or polymicrobial growth. Bacterial isolates were further classified as Gram-positive cocci, Gram-negative bacilli, or mixed flora. Specific organisms, including *Pseudomonas* *aeruginosa* and *Klebsiella spp.*, were recorded when identified. Fungal growth from ulcer specimens was also recorded when identified. As this was a retrospective study, the analysis was restricted to microbiological investigations documented in the available hospital records.

Documentation of clinical diagnoses

Peripheral neuropathy and osteomyelitis were recorded based on the diagnoses documented in the patients’ clinical and hospital records. Other clinical variables, including peripheral arterial disease, foot deformity, ulcer characteristics, and prior episodes of diabetic foot ulcer, were similarly extracted from the available medical records using the standardized data collection form. As the study was retrospective, the analysis relied on the diagnostic assessments and documentation available in the hospital records.

Outcome measures

The primary outcome was the prevalence of onychomycosis among patients with diabetic foot ulcers. Secondary outcomes included the distribution of onychomycosis according to demographic and clinical characteristics; its association with ulcer microbiological findings, including specific bacterial isolates such as *Pseudomonas spp.*, *Klebsiella spp*., and Gram-positive cocci; the presence of polymicrobial infection; and markers of disease severity, including osteomyelitis.

Statistical analysis

Statistical analysis was performed using IBM SPSS Statistics for Windows, Version 23.0 (IBM Corp., Armonk, NY, USA). Categorical variables were summarized as frequencies and percentages. Univariate binary logistic regression analysis was performed to evaluate the association between clinical and microbiological variables and the presence of onychomycosis. The results were expressed as ORs with 95% CIs. A two-sided p-value of <0.05 was considered statistically significant. 

Because this was a retrospective study based on routinely recorded clinical and laboratory information, missing or unavailable data were not imputed. Analyses were performed using the available documented data, while patients with incomplete nail examination or microbiological records were excluded according to the predefined eligibility criteria.

## Results

A total of 226 patients with diabetic foot ulcers were included in the study. Confirmed onychomycosis was identified in 85 (37.6%) patients, while 141 (62.4%) had no evidence of onychomycosis (Table 1).

**Table 1:** Prevalence of onychomycosis among patients with diabetic foot ulcers

Status	n (%)
Present	85 (37.6)
Absent	141 (62.4)

The prevalence of onychomycosis increased with age. Among patients with onychomycosis, 11 (12.9%) were aged ≤50 years, 19 (22.4%) were aged 51-60 years, 35 (41.2%) were aged 61-70 years, and 20 (23.5%) were aged >70 years. Onychomycosis was more common among male patients (54, 63.5%) than female patients (31, 36.5%) (Table 2).

**Table 2:** Demographic characteristics according to onychomycosis status

Variable	Total (N=226) n (%)	Onychomycosis (N=85) n (%)
Age ≤50 years	42 (18.6)	11 (12.9)
Age 51–60 years	61 (27.0)	19 (22.4)
Age 61–70 years	78 (34.5)	35 (41.2)
Age >70 years	45 (19.9)	20 (23.5)
Sex		
Male	132 (58.4)	54 (63.5)
Female	94 (41.6)	31 (36.5)

According to diabetes-related clinical characteristics, six (7.1%) patients with onychomycosis had diabetes duration <5 years, 19 (22.4%) had diabetes duration of five to 10 years, and 60 (70.6%) had diabetes duration >10 years. Regarding glycemic control, eight (9.4%) patients had HbA1c ≤7.0%, 34 (40.0%) had HbA1c 7.1-9.0%, and 43 (50.6%) had HbA1c >9.0%. Peripheral neuropathy was present in 73 (85.9%) patients with onychomycosis (Table 3).

**Table 3:** Clinical characteristics according to onychomycosis status

Variable	Total (N=226) n (%)	Onychomycosis (N=85) n (%)
Diabetes duration		
<5 years	28 (12.4)	6 (7.1)
5–10 years	63 (27.9)	19 (22.4)
>10 years	135 (59.7)	60 (70.6)
HbA1c		
≤7.0	36 (15.9)	8 (9.4)
7.1–9.0	98 (43.4)	34 (40.0)
>9.0	92 (40.7)	43 (50.6)
Neuropathy present	171 (75.7)	73 (85.9)
Neuropathy absent	55 (24.3)	12 (14.1)

Regarding ulcer microbiology, seven (8.2%) patients with onychomycosis had no microbial growth, 34 (40.0%) had monomicrobial cultures, and 44 (51.8%) had polymicrobial cultures. Gram-positive cocci were identified in 27 (31.8%) patients, whereas Gram-negative bacilli were isolated in 49 (57.6%). *Pseudomonas aeruginosa* and *Klebsiella spp.* were isolated in 30 (35.3%) and 19 (22.4%) patients, respectively. Osteomyelitis was present in 35 (41.2%) patients with onychomycosis (Table 4).

**Table 4:** Microbiological characteristics according to onychomycosis status

Variable	Total (N=226) n (%)	Onychomycosis (N=85) n (%)
Culture growth		
No growth	38 (16.8)	7 (8.2)
Monomicrobial	104 (46.0)	34 (40.0)
Polymicrobial	84 (37.2)	44 (51.8)
Gram-positive cocci		
Present	77 (34.1)	27 (31.8)
Absent	149 (65.9)	58 (68.2)
Gram-negative bacilli		
Present	112 (49.6)	49 (57.6)
Absent	114 (50.4)	36 (42.4)
Pseudomonas spp.		
Present	59 (26.1)	30 (35.3)
Absent	167 (73.9)	55 (64.7)
Klebsiella spp.		
Present	42 (18.6)	19 (22.4)
Absent	184 (81.4)	66 (77.6)
Osteomyelitis		
Present	67 (29.6)	35 (41.2)
Absent	159 (70.4)	50 (58.8)

On univariate binary logistic regression analysis, diabetes duration >10 years (OR: 2.30, 95% CI: 1.24-4.28; p=0.008), peripheral neuropathy (OR: 2.67, 95% CI: 1.33-5.34; p=0.005), polymicrobial infection (OR: 2.26, 95% CI: 1.30-3.91; p=0.004), *Pseudomonas aeruginosa* isolation (OR: 2.10, 95% CI: 1.14-3.86; p=0.017), and osteomyelitis (OR: 2.38, 95% CI: 1.33-4.26; p=0.003) were significantly associated with onychomycosis (Table 5).

**Table 5:** Univariate binary logistic regression analysis of factors associated with onychomycosis among patients with diabetic foot ulcers

Variable	Crude OR	95% CI	p-value
Male sex	1.40	0.79–2.47	0.24
Diabetes duration >10 years	2.30	1.24–4.28	0.008
HbA1c >9%	1.66	0.96–2.83	0.070
Peripheral neuropathy	2.67	1.33–5.34	0.005
Polymicrobial culture	2.26	1.30–3.91	0.004
Gram-negative bacilli	1.69	0.98–2.91	0.060
Pseudomonas aeruginosa	2.10	1.14–3.86	0.017
Klebsiella spp.	1.47	0.75–2.88	0.260
Osteomyelitis	2.38	1.33–4.26	0.003

## Discussion

This retrospective observational study evaluated the prevalence of onychomycosis among patients with diabetic foot ulcers and its association with clinical and microbiological characteristics [8]. Onychomycosis was identified in more than one-third of the study population (37.6%). The condition was more prevalent among older patients, those with a longer duration of diabetes, and those with poor glycemic control. In addition, peripheral neuropathy showed a significant association with onychomycosis, consistent with the established role of altered pressure distribution, loss of protective sensation, and repeated nail trauma in the diabetic foot [9]. Furthermore, polymicrobial ulcer infections, Gram-negative bacilli, particularly *Pseudomonas aeruginosa *and *Klebsiella spp*., and osteomyelitis were more frequently observed among patients with onychomycosis. These findings were further supported by the results of the univariate binary logistic regression analysis [10].

The prevalence of onychomycosis observed in the present study (37.6%) is at the higher end of the range reported in previous regional and international studies involving patients with diabetes mellitus. Impaired immune function, compromised microcirculation, and repetitive unrecognized trauma in neuropathic feet have been recognized as important risk factors for fungal nail infections [11]. Consistent with previous reports, older age and longer duration of diabetes were associated with a higher prevalence of onychomycosis, suggesting that prolonged metabolic dysfunction may increase susceptibility to fungal colonization and infection. Similarly, poor glycemic control was associated with a greater prevalence of onychomycosis in the present study. Hyperglycemia impairs neutrophil function and increases glucose concentrations within skin and nail tissues, creating a favorable environment for microbial growth. Peripheral neuropathy further contributes by reducing protective sensation, predisposing patients to repeated trauma and subsequent fungal nail infection [12].

The association between onychomycosis and polymicrobial ulcer infections, particularly those involving Gram-negative bacilli, supports the growing concept that fungal nail disease may influence the microbial ecology of chronic diabetic foot ulcers. Thickened, dystrophic nails may serve as a reservoir for microorganisms and facilitate bacterial colonization of adjacent tissues [13]. In the present study, *Pseudomonas aeruginosa* and *Klebsiella spp*. were more frequently isolated among patients with onychomycosis. These organisms are commonly associated with chronic, biofilm-forming diabetic foot infections and are recognized contributors to delayed wound healing and increased disease severity [14,15].

The present findings emphasize the importance of incorporating routine nail examination into the clinical assessment of patients with diabetic foot ulcers. Although vascular status and peripheral neuropathy are routinely evaluated during diabetic foot examinations, nail disorders are often overlooked [16]. Untreated onychomycosis may contribute to nail thickening, periungual skin injury, and disruption of the skin barrier, thereby facilitating secondary bacterial colonization and potentially increasing the risk of ulcer progression. Early recognition and appropriate management of onychomycosis may reduce the microbial burden, improve foot hygiene, and potentially decrease the risk of secondary infection and osteomyelitis [17].

Considering the microbiological findings of this study, antifungal therapy should be considered when clinically indicated as part of a comprehensive management strategy for diabetic foot ulcers. Treatment may include appropriate antifungal pharmacotherapy, mechanical debridement of dystrophic nails, pressure offloading, and patient education regarding foot hygiene to optimize overall foot care [17].

Strengths and limitations

The strengths of this study include the relatively large sample size of patients with diabetic foot ulcers and the use of microbiological confirmation of onychomycosis in addition to clinical assessment. Furthermore, a broad range of demographic, clinical, and microbiological variables was evaluated, allowing comprehensive assessment of factors associated with onychomycosis.

However, this study has several limitations. Its retrospective design relied on routinely recorded clinical and laboratory data, making it susceptible to missing information and potential misclassification. Nail microscopy and fungal culture were not performed in all patients, and the retrospective analysis therefore depended on the diagnostic investigations documented in the hospital records. This may have resulted in underestimation of the true prevalence of onychomycosis. In addition, follow-up data regarding antifungal treatment, ulcer healing, recurrence, and long-term clinical outcomes were unavailable. Finally, the observational nature of the study precludes the establishment of causal relationships.

Future directions

Prospective multicenter studies using standardized fungal diagnostic protocols and longitudinal follow-up are warranted to determine whether treatment of onychomycosis reduces ulcer recurrence, infection severity, and the need for surgical intervention. Incorporation of molecular diagnostic techniques may further improve the detection of mixed fungal-bacterial biofilms and enhance understanding of microbial interactions in diabetic foot ulcers.

## Conclusions

This study followed a cohort of diabetic subjects with foot ulcers. The overall frequency of onychomycosis in this population is notable. There was a tendency for infection to be more common in older antipodal subjects, with chronic type 2 diabetes, poor glycaemic control, and signs of peripheral neuropathy. Microbiological assessment revealed a relatively greater rate of polymicrobially infected ulcers and Gram-negative bacilli (*Pseudomonas aeruginosa* and *Klebsiella spp.*) in those subjects with onychomycosis. Collectively, these findings lead us to suspect that fungal nail disease may be an aetiological component of the microbial signature associated with diabetic foot infections, and perhaps more severe local complications, including osteomyelitis. We advocate for the identification and treatment of onychomycosis alongside comprehensive diabetic foot care. Early recognition of toenail disease through regular nail assessment may help mitigate the burden of secondary bacterial infection, inform ulcer prevention initiatives, and perhaps improve local tissue health. Prospective studies are needed to determine the effect of definitive antifungal treatment on ulcer healing trajectories and risk of subsequent or worsening infection.
